# Former smoking, but not active smoking, is associated with delirium in postoperative ICU patients: a matched case-control study

**DOI:** 10.3389/fpsyt.2024.1347071

**Published:** 2024-03-14

**Authors:** Maria Angeliki Komninou, Simon Egli, Aurelio Rossi, Jutta Ernst, Michael Krauthammer, Reto A. Schuepbach, Marcos Delgado, Jan Bartussek

**Affiliations:** ^1^ Institute of Intensive Care Medicine, University Hospital Zurich & University of Zurich, Zurich, Switzerland; ^2^ Center of Clinical Nursing Sciences, University Hospital Zurich, Zurich, Switzerland; ^3^ Department for Quantitative Biomedicine, University of Zurich, Zurich, Switzerland; ^4^ Department of Anesthesia and Intensive Care Medicine, Tiefenau Hospital, Insel Group. University of Bern, Bern, Switzerland

**Keywords:** smoking, nicotine, postoperative delirium (POD), ICU delirium, critical care medicine, case-control studies, withdrawal, risk factors

## Abstract

**Objective:**

To examine the relationship between current and former smoking and the occurrence of delirium in surgical Intensive Care Unit (ICU) patients.

**Methods:**

We conducted a single center, case-control study involving 244 delirious and 251 non-delirious patients that were admitted to our ICU between 2018 and 2022. Using propensity score analysis, we obtained 115 pairs of delirious and non-delirious patients matched for age and Simplified Acute Physiology Score II (SAPS II). Both groups of patients were further stratified into non-smokers, active smokers and former smokers, and logistic regression was performed to further investigate potential confounders.

**Results:**

Our study revealed a significant association between former smoking and the incidence of delirium in ICU patients, both in unmatched (adjusted odds ratio (OR): 1.82, 95% confidence interval (CI): 1.17-2.83) and matched cohorts (OR: 3.0, CI: 1.53-5.89). Active smoking did not demonstrate a significant difference in delirium incidence compared to non-smokers (unmatched OR = 0.98, CI: 0.62-1.53, matched OR = 1.05, CI: 0.55-2.0). Logistic regression analysis of the matched group confirmed former smoking as an independent risk factor for delirium, irrespective of other variables like surgical history (p = 0.010). Notably, also respiratory and vascular surgeries were associated with increased odds of delirium (respiratory: OR: 4.13, CI: 1.73-9.83; vascular: OR: 2.18, CI: 1.03-4.59). Medication analysis showed that while Ketamine and Midazolam usage did not significantly correlate with delirium, Morphine use was linked to a decreased likelihood (OR: 0.27, 95% CI: 0.13-0.55).

**Discussion:**

Nicotine’s complex neuropharmacological impact on the brain is still not fully understood, especially its short-term and long-term implications for critically ill patients. Although our retrospective study cannot establish causality, our findings suggest that smoking may induce structural changes in the brain, potentially heightening the risk of postoperative delirium. Intriguingly, this effect seems to be obscured in active smokers, potentially due to the recognized neuroprotective properties of nicotine. Our results motivate future prospective studies, the results of which hold the potential to substantially impact risk assessment procedures for surgeries.

## 1 Introduction

Postoperative delirium, a neuropsychiatric syndrome characterized by sudden confusion, altered consciousness, and attention deficits, is a prevalent condition among hospitalized patients, with incidence rates ranging between 11% and 56% and escalating to 73% in Intensive Care Units (ICUs) (1–6). It not only prolongs hospitalization duration, morbidity and mortality, but also increases the risk of post-discharge complications, such as dementia (7). Delirium management in ICUs is associated with significantly higher healthcare expenditures (8, 9).

Numerous precipitating events and predisposing factors have been implicated in delirium onset, including older age, cognitive impairment, functional and sensory deficits, infections, physiological irregularities, pre-existing health conditions, medications, surgical procedures, and excessive alcohol consumption (10–13). Despite its high prevalence in ICU settings, the underlying pathophysiology of delirium remains largely elusive. Current theories propose that delirium may arise from an imbalance in neurotransmitter activity triggered by specific diseases, neuroinflammatory processes or therapeutic interventions. These disrupt normal brain functioning, network connectivity and trigger disorientation, compromised attention, and consciousness fluctuations (14). This neurochemical imbalance may involve elevated levels of neurotransmitters like dopamine, glutamate, and norepinephrine, as well as alterations in gamma-aminobutyric acid (GABA), histamine, serotonin, and acetylcholine, the latter being critical for memory and learning processes (15).

While various factors associated with delirium incidence have been investigated, the role of smoking in the pathogenesis of delirium has received limited attention. Existing data on smoking and delirium show inconsistency due to a variety of factors such as variations in study design, differing methods of controlling for confounders, or diverse patient populations across studies. Some studies suggest an increased risk of hospital-related delirium in smokers (16–18), while others report no significant impact (19–22), and some findings remain inconclusive (23, 24). Nicotine, an active component of tobacco, mimics the function of acetylcholine by binding to specific nicotinic cholinergic receptors (nAChRs), leading to the release of various neurotransmitters including dopamine, serotonin, glutamate, GABA, and noradrenaline. These enable enhanced alertness, attention, cognitive performance, relaxation, and stress reduction (25, 26).

Given the complex neuropharmacological effects of nicotine and the limited attention given to the role of smoking in delirium pathogenesis, it is essential to further investigate the impact of smoking on delirium risk. Distinguishing between active and former smokers can help identify long-term effects on the brain from recent smoking or nicotine withdrawal impacts. Therefore, our study aims to explore the relationship between smoking status – active and former – and the risk of delirium in ICU patients. By analyzing both unmatched and matched patient groups, we aim to provide a comprehensive perspective on how smoking status affects delirium risk in our study population.

## 2 Methods

### 2.1 Study design

**Figure 1:**
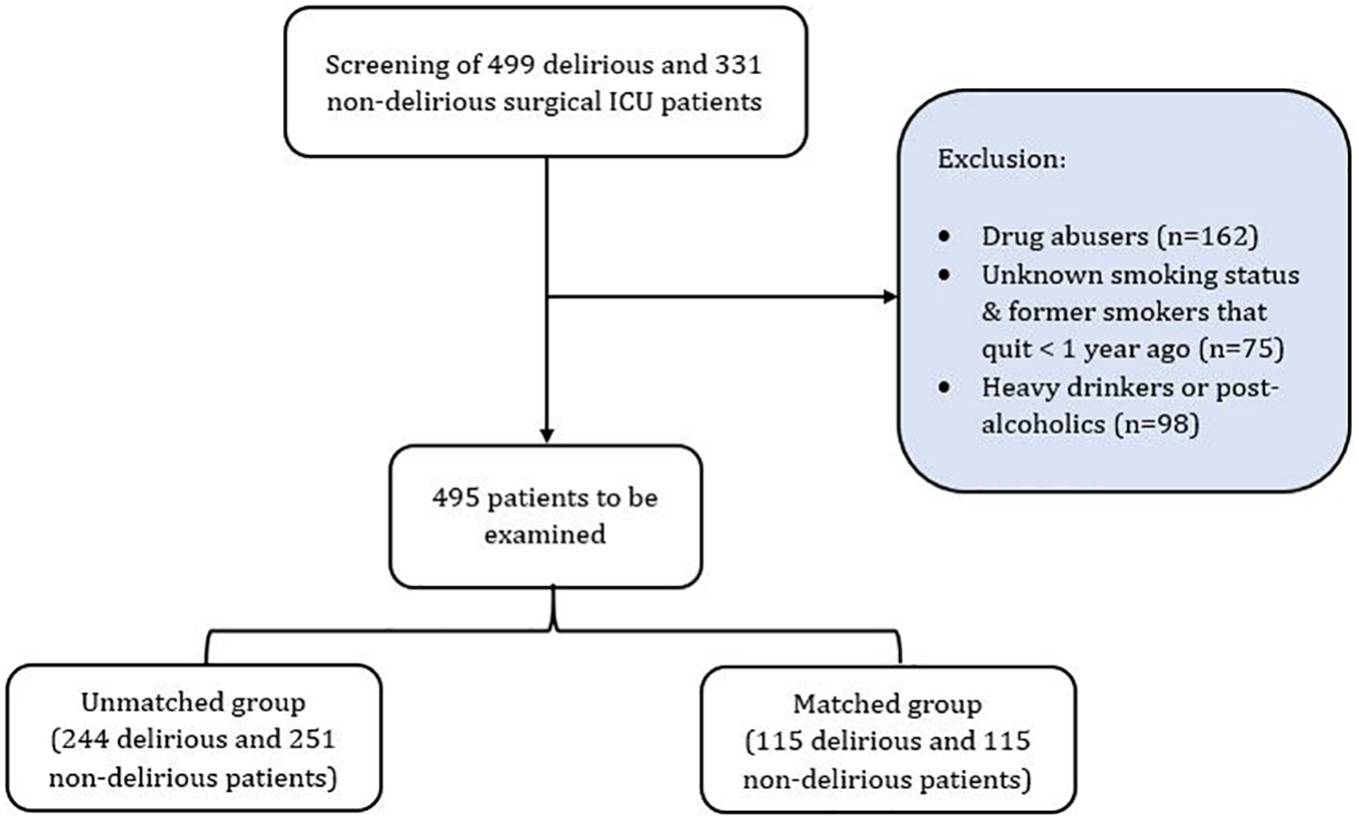
Patient selection, exclusion criteria and grouping process for our study.

In a retrospective study, data from patients admitted to the Intensive Care Unit of the University Hospital Zurich’s Institute of Intensive Care Medicine were scrutinized (**Figure 1**). Patients being admitted to the ICU between 1^st^ of January 2018 and 30^th^ of January 2022 were included in the research. Inclusion criteria entailed (i) an absence of documented objection to data utilization, (ii) an age exceeding 18 years and (iii) documented ICDSC (Intensive Care Delirium Screening Checklist) scores. Exclusion criteria included (i) lack of data regarding smoking status, (ii) cessation of smoking less than 1 year prior, (iii) unknown delirium status, (iv) drug abuse, (v) current or former alcohol abuse. Delirium status during ICU stay was determined using a combination of ICD-10 diagnosis code (27) and ICDSC score. The ICDSC scoring system ranges from 0 to 8, with a score of 4 or higher serving as the threshold for delirium (28). Patients with a maximal ICDSC score of 4 or above, coupled with an ICD-10 diagnosis of delirium (code F05), were categorized as delirium cases (D). Conversely, those with ICDSC scores below 4 and no ICD-10 diagnosis of delirium were categorized as controls (ND). From all patients that fulfilled the criteria (n=495), 230 were matched for age and SAPS II.

### 2.2 Smoking status

The smoking status of patients was determined through the pre-ops anesthesia anamnesis in the form of pack-years (the unit of measurement used to quantify the level of exposure to cigarette smoking, calculated by multiplying the number of packs of cigarettes smoked per day by the number of years the person has smoked) (29) and smoking status. Smoking status was encoded in the anamnesis as “non-smokers” (patients who reported no history of smoking), “active-smokers” (those who were currently smoking), “former smokers” (patients who had quit smoking for at least one year) and “quitters” (patients who reported smoking cessation within the past year).

### 2.3 Confounding factors

To minimize the influence of confounding variables, we excluded “quitters” from the study to account for potential lingering effects of nicotine (30) and the persistence of tobacco abstinence symptoms (31). Moreover, we aimed to mitigate the potential confounding effects of alcohol consumption on our results. Alcohol use disorder (AUD) is a significant risk factor for delirium tremens (32) and often accompanied with heavy smoking. To ensure that any observed effects were not primarily driven by the detrimental impact of excessive alcohol intake on the brain (33, 34), we restricted our study population to patients with zero, mild (a few glasses of alcohol per month), or moderate alcohol consumption (a few glasses of alcohol per week), while individuals with a history of alcoholism or heavy drinking (men: more than 2 glasses of alcohol per day, women: more than 1 glass of alcohol per day) were excluded. The applied definition alcohol consumption adhered to our Intensive Care Unit’s guidelines.As an additional consideration for potential confounding factors, we evaluated the consumption of medications known to affect the central nervous system, including opioids, benzodiazepines, and alpha agonists (35–38). These medication categories, commonly prescribed in a hospital setting, particularly opioids and benzodiazepines, have been associated with an increased risk of delirium (39).

To uniformly compare medication use across patients, we standardized the analysis timeframe. For patients who developed delirium, we examined medication data from the 72 hours prior the first positive ICDSC score. Conversely, for non-delirious patients, we analyzed the medication data in the 72-hour window preceding their last ICDSC assessment prior to ICU discharge. This consistent approach in timeframe selection ensured comparability in our longitudinal medication data analysis (40).

Given the established associations between delirium and mechanical ventilation (41), pain (42) and cognitive decline (43), we also examined the prevalence of these confounding factors in our study. For pain, we pooled the results of the standardized nursing assessments (NANDA) including both numerical rating scales and verbal rating scales for pain. For cognitive decline, we pooled ICD-10 diagnoses like delirium superimposed on dementia (F05.1), vascular dementia (F01.8), unspecified dementia (F03), mild cognitive disorder (F06.7) and unspecified mental retardation (F79.9 - without mention of impairment of behavior). Additionally, the most common primary diagnoses and CHOP codes (Swiss operation classification) codes were analyzed.

### 2.4 Statistical analysis

Advanced age and illness severity are recognized as significant risk factors for delirium (44–46). To mitigate the confounding influences of these variables in our analysis, we utilized the propensity score matching technique. To this end, we conducted logistic regression for all 495 participants to generate propensity scores, which estimate the probability of delirium occurrence, based on age and the SAPS II score (a proxy for illness severity) as the key variables for matching. Subsequently, we employed the nearest neighbor matching strategy to pair individuals with delirium (treated group) with those without delirium (control group) who had the closest propensity scores. This approach resulted in the creation of 115 matched pairs, substantially reducing the confounding effects of age and illness severity on our study outcomes.

Descriptive statistics were calculated for each variable of interest, including investigating pack-years, medications’ doses, mechanical ventilation, most common reasons of admission, and most frequently performed surgeries and afterwards, we stratified our delirious and non-delirious data by smoking status to further investigate its effect.

T-tests and One-way ANOVA tests were performed to evaluate the association between the continuous variables and delirium incidence and odds ratios were calculated to estimate the strength of these associations. Chi-square tests and exact Fisher’s tests were applied for categorical variables, the investigation of medication use and delirium incidence. Finally, logistic regression was performed to investigate the variables with remaining significance.

All statistical analyses were performed using the Scientific Python Development Environment Spyder IDE (Python 3.9.7 64-bit), and a p ≤ 0.05 was considered statistically significant.

## 3 Results

In this study, we initially included 244 patients with delirium (D) and 251 without delirium (ND), as shown in **Table 1**A. To control for confounding factors, we implemented a matching strategy. By aligning patients based on their age and SAPS II scores, we obtained two balanced groups comprising 115 patients each.

**Table 1:** A: Statistical analysis of demographical and clinical characteristics. B: Comparison of most common primary diagnoses and surgical categories.

A							
Unmatched group				Matched group			
Description	D	ND	p-value	Description	D	ND	p-value
N of total patients	244	251	–	N of total patients	115	115	–
Females, n (%)	74 (30.3)	93 (37.0)	0.050	Females, n (%)	33 (28.7)	42 (36.5)	0.191
Age, mean/median (IQR)	66.2/68.0 (19.0)	57.3/60.0 (23.0)	1.200 x 10^-9^	Age, mean/median (IQR)	62.3/65.0 (22.0)	63.1/65.0 (19.0)	0.686
SAPS II, mean/median (IQR)	49.8/48.0 (19.0)	37.6/37.0 (21.0)	1.500 x 10^-15^	SAPS II, mean/median (IQR)	44.6/45.0 (23.0)	44.3/43.0 (21.5)	0.878
Mech. ventilation, n (%)	232 (95.1)	214 (85.2)	0.255	Mech. ventilation, n (%)	110 (95.6)	102 (88.7)	0.500
Pain, n (%)	27 (11.2)	28 (11.2)	1.0	Pain, n (%)	7 (6.1)	9 (7.8)	0.724
Cognitive impairment, n (%)	16 (6.6)	6 (2.4)	0.007	Cognitive impairment, n (%)	6 (5.2)	4 (3.5)	0.655
B							
Unmatched group				Matched group			
Most common diagnosis categories, n	D	ND	p-value	Most common diagnosis categories, n	D	ND	p-value
Circulatory conditions	106	102	0.769	Circulatory conditions	62	52	0.233
Injuries, poisoning & burns	32	24	0.186	Neoplasms	16	29	0.011
Neoplasms	29	62	2.100 x 10^-6^	Respiratory system	12	7	0.194
Respiratory system	28	22	0.317	Injuries, poisoning & burns	10	7	0.493
Infectious and parasitic diseases	16	5	0.002	Infectious and parasitic diseases	6	4	0.655
Unmatched group				Matched group			
Most common surgeries^*^, n	D	ND	p-value	Most common surgeries, n	D	ND	p-value
Vascular	218	150	7.800 x 10^-7^	Vascular	105	77	0.005
Gastrointestinal	184	100	3.280 x 10^-12^	Cardio	77	66	0.237
Cardio	145	136	0.158	Gastrointestinal	67	54	0.123
Respiratory	121	47	1.650 x 10^-15^	Respiratory	53	16	8.840 x 10^-10^
Integumentary	66	30	4.380 x 10^-7^	Nervous	31	40	0.179

D, delirious; ND, non-delirious; IQR, Interquartile range; SAPS II, Simplified Acute Physiology score II; *patients can have multiple surgeries.

The matched patient population showed a comparable distribution of sex across the D and ND groups (p = 0.191). This consistency extended to age and SAPS II scores post-matching, where no significant differences were found (p = 0.686 and p = 0.878, respectively), indicating successful alignment of mean and median ages, as well as illness severity between groups. Additionally, the utilization of mechanical ventilation showed no significant variance between D and ND patients in either unmatched (p = 0.255) or matched cohorts (p = 0.500), and similarly, among patients that experienced pain, no significant difference between D and ND patients in either unmatched (p = 1.0) or matched cohorts (p = 0.724) was observed. Regarding the cognitive impairment, a significant difference between D and ND patients was observed in the unmatched group (p = 0.007), that was eventually lost when groups were matched (p = 0.655).

Our comparison of the most common ICD-10 diagnosis categories and surgical procedures between delirious and non-delirious patients considered both unmatched and matched patient data (**Table 1**B). In the unmatched cohort, notable differences emerged. Specifically, non-delirious patients exhibited a higher incidence of neoplasms (p = 2.1 x 10^6^), while delirious patients had a greater prevalence of infectious and parasitic diseases (p = 0.002). Apart from cardiac surgeries, all surgical categories differed significantly between D and ND patients in the unmatched data.

Post-matching, the higher incidence of neoplasms in non-delirious patients persisted as a significant finding (p = 0.011). Furthermore, vascular and respiratory surgeries continued to show a higher frequency in delirious patients (p = 0.005 and p = 8.84 x 10^-10^, respectively). However, other diagnosis categories and surgical procedures did not demonstrate significant differences in the matched cohorts, underscoring the effectiveness of our matching strategy in controlling for confounding variables.

Following our assessment of patient demographics, we analyzed the smoking status in relation to delirium. Chi-square tests were used to compare the proportions of non-smokers, active smokers, and former smokers between D and ND groups. This analysis included both unmatched and matched cohorts, as detailed in **Table 2**.

**Table 2:** Smoking status and pack-years for delirious (D) and non-delirious (ND) patients for both total (unmatched) and matched patients. .

	Total (unmatched) patients							Matched patients						
	Delirious			Non-delirious			p-value	Delirious			Non-delirious			p-value
Status	NS	AS	FS	NS	AS	FS		NS	AS	FS	NS	AS	FS	
Activity, n (%)	124 (50.8)	49 (20.1)	71 (29.1)	146 (58.2)	59 (23.5)	46 (18.3)	0.020	52 (45.2)	24 (20.9)	39 (33.9)	68 (59.1)	30 (26.1)	17 (14.8)	0.003
Pack-years (median)	–	30.0	30.0	–	20.0	30.0	0.080	–	20.0	20.0	–	22.5	38.0	0.270
***Post-hoc* analysis**				**Group**		**p-value**		***Post-hoc* analysis**			**Group**		**p-value**	
				NS-AS		1.0					NS-AS		1.0	
				AS-FS		0.020					AS-FS		0.060	
				NS-FS		0.010					NS-FS		0.001	

D, delirious; ND, non-delirious; NS, non-smokers; AS, active smokers; FS, former smokers.

Prior to matching, a higher proportion of non-delirious patients were non-smokers (58.2%) compared to their delirious counterparts (50.8%). Active smoking was somewhat more prevalent in the ND group (23.5%) than in the D group (20.1%). However, the most notable difference was observed in former smokers, where 29.1% of delirious patients were former smokers, in contrast to 18.3% in the ND group. Overall, disparity in smoking status between the two groups was statistically significant (p = 0.020).

After matching, disparities in smoking status between delirious (D) and non-delirious (ND) patients became more pronounced (p = 0.003). In the matched cohort, the ND group had a higher proportion of non-smokers (59.1%) and active smokers (26.1%), but a smaller proportion of former smokers (14.8%) compared to the D group, which comprised 45.2% non-smokers, 20.9% active smokers, and 33.9% former smokers.

*Post-hoc* analyses within each group underscored no significant differences between non-smokers and active smokers (p = 1.0 in both unmatched and matched groups). However, significant distinctions were evident between active smokers and former smokers (p = 0.020 in the unmatched group and 0.060 in the matched group), as well as between non-smokers and former smokers (p = 0.010 in the unmatched group and 0.001 in the matched group). These findings highlight a notably higher prevalence of delirium among former smokers.

When examining median pack-years, a measure indicative of cumulative smoking exposure, we found no significant differences between delirious and non-delirious patients in both unmatched (p = 0.080) and matched groups (p = 0.270).

**Figure 2:**
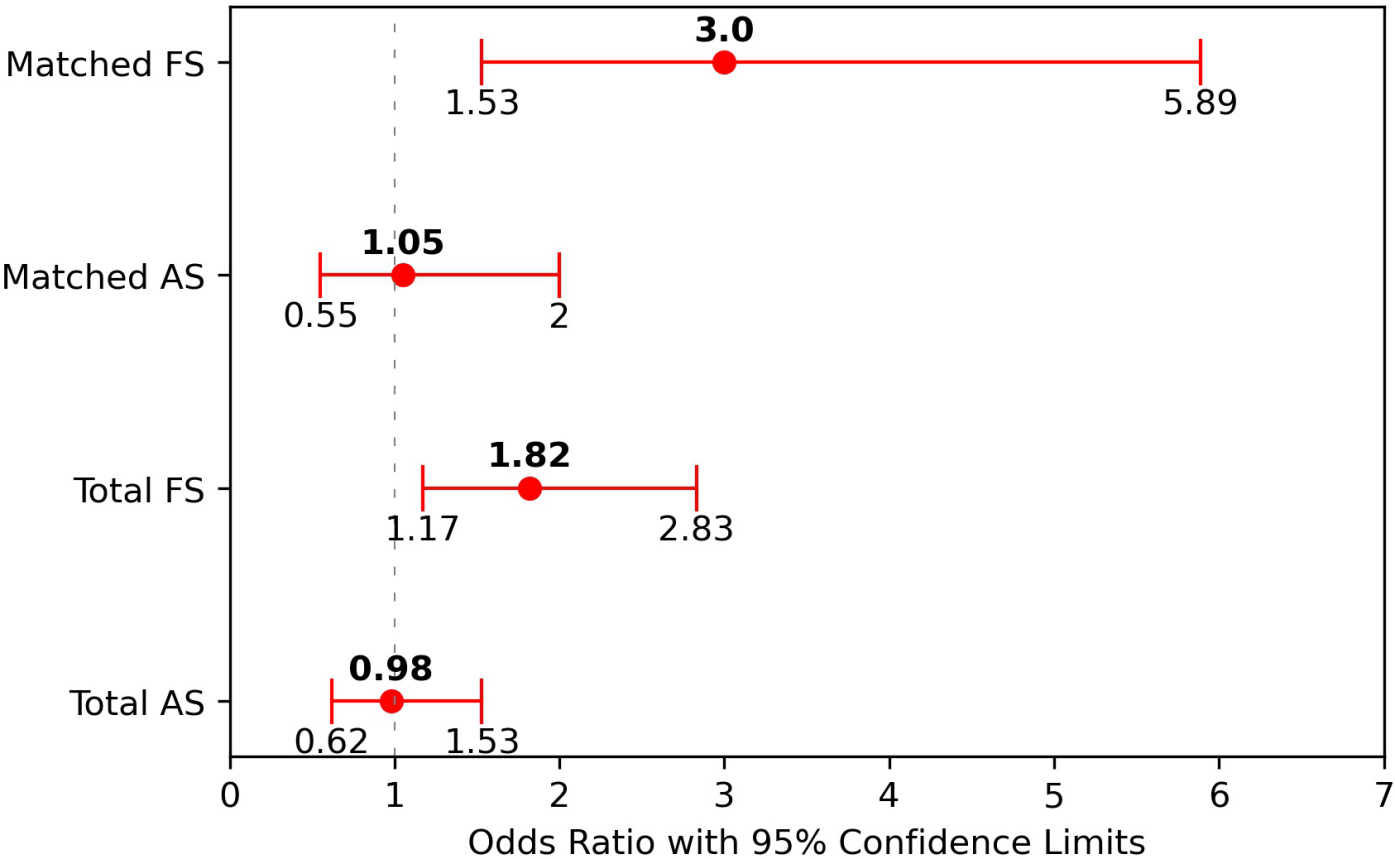
Odds ratio (OR) and 95% confidence intervals (CI) for active and former smokers for both total and matched patients. The odds ratios refer to active smokers (AS) and former smokers (FS) against the non-smokers.

In order to quantify the strength of association between smoking status and delirium, we calculated the odds ratios (OR) and confidence intervals (CI) in both the total (unmatched) and matched patient populations (**Figure 2**). Former smokers showed a significantly elevated risk of delirium compared to non-smokers with OR of 1.82 (95% CI: 1.17-2.83) in the total population and 3.0 (95% CI: 1.53-5.89) in the matched analysis. The odds ratio analysis comparing active smokers to non-smokers did not reveal a significant difference in delirium risk (unmatched OR = 0.98, 95% CI: 0.62-1.53 and matched OR = 1.05, 95% CI: 0.55-2.0).

To assess the potential confounding impact of analgesics and sedatives, known risk factors for delirium, on our smoking-related findings, we conducted a comparative analysis of medication use (**Table 3**). In the unmatched group, a significant difference was observed in Fentanyl administration, with 41.0% in delirious patients compared to 51.4% in non-delirious patients (p = 0.010). Ketamine and Midazolam were significantly more prevalent in delirious patients (p = 0.020 and p = 0.006, respectively), while Morphine usage was higher in the non-delirious group (44.6% vs. 29.1%, p = 2.9 x 10^-5^). No significant differences were found for Sufentanil, Propofol, and Dexmedetomidine, with p-values of 0.370, 0.738, and 0.123, respectively. In the matched cohort, the significance observed pre-matching persisted for Ketamine (p = 0.002), Morphine (p = 0.001), and Midazolam (p = 0.0003).

**Table 3:** Comparison of medication administration for delirious (D) and non-delirious (ND) patients.

	Unmatched group, n(%)			Matched group, n(%)		
Medication	D	ND	p-value	D	ND	p-value
Fentanyl^1^	100 (41.0)	129 (51.4)	0.01	53 (46.1)	61 (53.0)	0.354
Ketamine^1^	38 (15.6)	24 (9.6)	0.02	24 (20.9)	10 (8.7)	0.002
Morphine^1^	71 (29.1)	112 (44.6)	2.9 x 10^-5^	36 (31.3)	59 (51.3)	0.001
Sufentanil^1^	84 (34.4)	75 (29.9)	0.370	47 (48.9)	40 (34.8)	0.363
Midazolam^2^	58 (23.8)	38 (15.1)	0.006	37 (32.2)	17 (14.8)	0.0003
Propofol^3^	141 (57.8)	146 (58.2)	0.738	78 (67.8)	70 (60.9)	0.416
Dexmedetomidine^4^	57 (23.4)	45 (17.9)	0.123	34 (29.6)	29 (25.2)	0.476

D, delirious; ND, non-delirious; ^1^ Opioids; ^2^ Benzodiazepines; ^3^ Non-barbiturate sedatives, ^4^ Alpha-agonists.

To confirm the independent association of smoking status as a risk factor for delirium, we performed logistic regression analysis including all variables that demonstrated significant differences, such as various medications and surgical histories (**Table 4**). The analysis revealed that being a former smoker significantly increased the odds of experiencing delirium (p = 0.010, OR: 2.87, 95% CI: [1.29, 6.37]). Additionally, patients undergoing respiratory or vascular surgeries were found to have higher odds of delirium (respiratory: p = 0.001, OR: 4.13, 95% CI: [1.73, 9.83]; vascular: p = 0.040, OR: 2.18, 95% CI: [1.03, 4.59]). Contrarily, the use of Ketamine or Midazolam did not show a significant association with delirium (p = 0.269 and 0.117, respectively), while Morphine use was linked to a decreased likelihood of delirium (p < 0.001, OR: 0.27, 95% CI: [0.13, 0.55]).

**Table 4:** Logistic regression for variables with remaining significance after matching.

Variable	Coefficient	Standard Error	z-value	p-value	OR	95% CI
Const	-0.4752	0.369	-1.289	0.197	0.62	[0.30, 1.28]
Former Smokers (Y/N)	1.0546	0.407	2.591	0.010	2.87	[1.29, 6.37]
Ketamine status (Y/N)	0.5118	0.463	1.104	0.269	1.67	[0.67, 4.14]
Morphine status (Y/N)	-1.3194	0.370	-3.566	0.000	0.27	[0.13, 0.55]
Midazolam status (Y/N)	0.5981	0.381	1.569	0.117	1.82	[0.86, 3.84]
Vascular surgery (Y/N)	0.7790	0.380	2.049	0.040	2.18	[1.03, 4.59]
Respiratory surgery (Y/N)	1.4179	0.442	3.206	0.001	4.13	[1.73, 9.83]

## 4 Discussion

Our study offers a novel perspective on the complex relationship between smoking history and the incidence of delirium, By isolating former smokers as a distinct group, we aimed to elucidate their specific risk of delirium, a departure from previous studies that often combined former smokers with either current or non-smokers (16, 20). Our findings revealed a significant link between former smoking and delirium across both unmatched and matched patient populations, suggesting the broad relevance of this relationship within the intensive care unit (ICU) setting. Contrary to prior research that reported inconclusive associations between smoking and delirium (10, 24), our approach sheds light on the potential impact of smoking history on neurological outcomes. The cognitive impairments attributed to chronic nicotine exposure, such as receptor up-regulation and tolerance (47), may contribute to the observed link between former smoking and delirium. This assertion aligns with the work of Anstey et al. (48), which links accelerated cognitive decline in former smokers to neurovascular damage and changes in gray matter. Our observations suggest that past smoking history could indeed influence neurological outcomes like delirium (49, 50). In this direction, a recent study unveils that smoking not only affects immune responses in the short term but also induces lasting changes in the body’s defense mechanisms (51). Specifically, it was found that the effects of smoking on adaptive immunity can persist for 10 to 15 years after cessation, a phenomenon attributed to epigenetic changes in DNA methylation affecting gene expression involved in immune cell metabolism.

This insight into the durable influence of smoking on the immune system provides a possible explanation for the increased susceptibility of former smokers to delirium in the ICU setting, as neuroinflammation is a potential mechanism of POD (52). Hence, epigenetic alterations resulting from past smoking may alter neurological outcomes by influencing the body’s inflammatory response and immune cell activity.

In contrast, our study did not demonstrate a similar difference in delirium incidence between active smokers and non-smokers. The existing literature on the relationship between active smoking and the development of delirium presents mixed findings. While some studies have pointed to a negative impact of smoking on delirium risk (16–18, 53, 54), others have yielded inconclusive results (23, 24) or even proposed that smoking might have a preventive effect against the onset of delirium (19).

A preventive effect of active smoking might elucidate how smoking-related alterations in brain structure and epigenetic modifications could lead to an elevated risk in former smokers, but not in current smokers. In this scenario, the harmful impacts of smoking are obscured by its preventive benefits. Nicotine and its metabolites, such as cotinine, are recognized for their anti-inflammatory properties, which may confer neuroprotective effects (55). Such properties may shield against oxidative stress-induced neuronal damage (56), particularly in regions such as the hippocampus (57), and provide protection against POD through the cholinergic anti-inflammatory pathway involving the vagus nerve and acetylcholine (19). The discussion of smoking history and its impact on neurological outcomes extends beyond delirium to diseases such as Parkinson’s disease (PD) and Alzheimer’s disease. Barreto et al.’s research demonstrates that nicotine and certain derivatives can mitigate oxidative stress and neuroinflammation in the brain, enhancing synaptic plasticity and supporting the survival of dopaminergic neurons (55). Furthermore, longitudinal studies suggest a protective effect of smoking on PD, with active smokers exhibiting a lower risk compared to never smokers, a trend that diminishes with increasing time since quitting (58). Gao et al. investigated the neuroprotective effects of cotinine and its analogs, including their comparison to existing Alzheimer’s disease therapies, highlighting cotinine’s potential as a superior therapeutic agent due to its longer half-life, lower toxicity, and lack of abuse potential, against the backdrop of Alzheimer’s complex pathophysiology and the limitations of current treatments (59).

After we matched our patients for age and SAPS II, also other confounding factors such as sex, mechanical ventilation, pain and cognitive impairement did not significantly differ between delirious and non-delirious groups. In the context of diseases and surgeries, significant disparities in ICD-10 diagnosis categories remained, suggesting a complex relationship between disease pathology and cognitive outcomes (60, 61). Thenotable link between delirium and vascular or respiratory surgeries—deviating from the usual association with cardiac procedures—may stem from the stress and systemic inflammation these surgeries induce (62, 63). The complexity and duration of such surgeries increase risks like hemodynamic instability and extensive blood loss (64, 65), while respiratory surgeries specifically risk postoperative hypoxemia, affecting cerebral oxygenation and elevating delirium risk (66). Vascular surgeries may also cause endothelial damage and pro-inflammatory cytokine release, possibly disrupting the blood-brain barrier (67). These findings highlight the need for vigilant delirium screening and specialized care for high-risk surgery patients to mitigate delirium risks.

Finally, our examination of medication use and the occurrence of delirium revealed a nuanced landscape. Despite the known associations of opioids and benzodiazepines with delirium in ICUs, our findings did not corroborate this (50, 68). Intriguingly, morphine administration was correlated with a reduced incidence of delirium as proven by the logistic regression, and ketamine use was not significantly linked to delirium, suggesting a protective analgesic role against the condition (69). This perspective is supported by research indicating that unmanaged pain is a contributory factor to delirium development, and thus, interventions that alleviate pain could indirectly reduce delirium incidence (70). However, it contradicts Duprey et al.’s conclusions, which implicated these medications in the development of delirium within intensive care settings (71). Lastly, it is important to note that our investigation’s scope did not extend to the impact of specific drugs or their temporal relationship with the onset of delirium, pointing to potential avenues for future research.

### 4.1 Limitations of the study

The present study carries some inherent limitations to be acknowledged: Its retrospective design may introduce inaccuracies or omissions in the gathered data. Furthermore, self-reported information from participants regarding their smoking and drinking habits might be inaccurate. While efforts were made to collect accurate data, the possibility of intentional misreporting cannot be completely excluded. Additionally, our strategy to match participants based on specific variables such as age and SAPS II, while beneficial in mitigating potential confounders, may limit the generalizability of our findings to populations that exhibit differences in these parameters. Moreover, the relatively modest sample size of our study may curtail the statistical power of our analyses, thereby escalating the probability of a type II error. Furthermore, we acknowledge that our dichotomous medication analysis does not account for potential dose-dependent effects of sedative and analgesic medications on delirium risk that could further elucidate the complex dynamics between medication dosage, administration methods, and delirium risk in postoperative ICU patients. Finally, our decision to exclude individuals who ceased smoking less than a year prior to the study could introduce a selection bias. Such excluded patients might have differing characteristics or outcomes compared to those incorporated in the study, potentially affecting our results. Regardless of these limitations, we believe our findings provide valuable insights into the relationship between smoking status and delirium, warranting further exploration in future studies.

## 5 Conclusion

This retrospective analysis does not support a link between active smoking and delirium in ICU patients. However, it reveals an association between former smoking and increased delirium risk. These findings underscore the importance of differentiating between current and former smoking statuses, as both may have beneficial or harmful effects that can occur either acutely or with a delay. Moving forward, researchers should prioritize investigating the underlying mechanisms behind this association, with a focus on developing risk-reducing strategies.

## Data availability statement

The raw data supporting the conclusions of this article will be made available by the authors upon request.

## Ethics statement

The studies involving humans were approved by Kantonale Ethikkommission Zürich (BASEC 2020-02695). The studies were conducted in accordance with the local legislation and institutional requirements. Written informed consent for participation was not required from the participants or the participants’ legal guardians/next of kin in accordance with the national legislation and institutional requirements.

## Author contributions

MK: Data curation, Formal Analysis, Validation, Visualization, Writing – original draft, Writing – review & editing. SE: Investigation, Writing – original draft, Writing – review & editing. AR: Investigation, Writing – review & editing. JE: Writing – review & editing. MK: Funding acquisition, Resources, Writing – review & editing. RS: Conceptualization, Funding acquisition, Methodology, Project administration, Resources, Supervision, Writing – review & editing. MD: Conceptualization, Methodology, Writing – original draft, Writing – review & editing. JB: Conceptualization, Funding acquisition, Investigation, Methodology, Project administration, Resources, Supervision, Writing – review & editing.
